# Disproportionately Elevated Proinsulin Levels as an Early Indicator of *β*-Cell Dysfunction in Nondiabetic Offspring of Chinese Diabetic Patients

**DOI:** 10.1155/2016/4740678

**Published:** 2016-09-25

**Authors:** Ming Li, Dan Feng, Kui Zhang, Shan Gao, Juming Lu

**Affiliations:** ^1^Department of Endocrinology, Key Laboratory of Endocrinology, National Health and Family Planning Commission, Peking Union Medical College Hospital, Chinese Academy of Medical Sciences, Beijing 100730, China; ^2^Department of Endocrinology, Beijing Chaoyang Hospital, Capital Medical University, 5 Jingyuanlu, Beijing 100043, China; ^3^Department of Endocrinology, PLA General Hospital, Beijing 100853, China

## Abstract

*Objective.* To study the characteristics of *β*-cell dysfunction and insulin resistance (IR) in the first-degree relatives (FDRs) of T2DM in Chinese population and to examine the usefulness of proinsulin (PI) for evaluating *β*-cell dysfunction.* Methods*. 229 subjects of nondiabetic FDRs, 71 newly diagnosed T2DM, and 114 with normal glucose tolerance (NGT) but not FDRs (NGT-non-FDRs) were verified by a 2-hour oral glucose tolerance test. Specific insulin (SI) and PI were measured by highly sensitive ELISA.* Results*. Compared to NGT-non-FDRs, NGT-FDRs showed higher levels of fasting and 2-hour PI, fasting PI-to-SI ratio (FPI/SI), and HOMA-IR (*p* < 0.01). Meanwhile, fasting PI, FPI/SI, and HOMA-IR were increased steadily from NGT-FDRs to prediabetes-FDRs and were highest in T2DM group (*p* < 0.001), whereas a significant decrease in HOMA-B could be observed only in T2DM group. Moreover, a progressive deterioration of *β*-cell function in NGT-FDRs, prediabetes-FDRs, and T2DM could be identified by FPI/SI even after adjusting for HOMA-IR: relative to non-FDRs controls, mean FPI/SI levels were increased 1.5, 2.0, and 4.7-fold, respectively (all *p* < 0.01).* Conclusions*.  *β*-cell dysfunction as assessed by disproportionate secretion of proinsulin and IR by HOMA (using specific insulin assay) already exist in FDRs of T2DM even with normal glucose status. Compared with HOMA-B, FPI/SI could detect *β*-cell failure in earlier stage of diabetes development.

## 1. Background 

Insulin resistance (IR) and *β*-cell dysfunction play the major pathophysiologic roles in type 2 diabetes mellitus (T2DM) [1]. Assessment of IR and *β*-cell dysfunction is essential not only to predict diabetes onset and progression but also to improve prevention and therapeutic strategies [2]. Euglycemic hyperinsulinemic clamp and hyperglycemic clamp are often referred to as “gold standard” [3], but they are not feasible in large-scale clinical trials or epidemiological studies. Some surrogate markers have been developed subsequently [4–7]; the most common index was the homeostasis model assessment (HOMA). However, HOMA was not so accurate, as it was highly affected by the technical limitations of conventional insulin assays: the cross-reactivity with proinsulin and lack of acceptable sensitivity to measure low fasting insulin levels; hence, its performance depends on insulin assay and could be improved by using highly sensitive and specific method [8–10]. On the other hand, with the availability of specific method to measure proinsulin, it has been shown that the disproportionate secretion of proinsulin, the precursor of insulin, can be not only a specific indicator of IR but also a hallmark of *β*-cell dysfunction [11, 12]; therefore, circulation proinsulin levels as well as proinsulin-to-insulin ratios have become the valuable measures to assess the impact of therapeutic interventions on the improvement of islet insulin-secreting function [13–15] and were somewhat better than HOMA indices. However, studies in Chinses population are lacking.

Notably, although the general consensus is that both IR and *β*-cell dysfunction are essential components in the development of T2DM, recent genome-wide association studies (GWAS) have discovered that the great majority of genetic variants conferring risk for T2DM are associated with *β*-cell function, implicating that *β*-cell dysfunction rather than insulin sensitivity might be relatively more important in T2DM pathogenesis [2, 16, 17]. In addition, studies in Asian patients have reported that diabetes develops at a lower mean body mass index (BMI) compared with those of European descent and is characterized by early *β*-cell dysfunction in the setting of IR, indicating ethnic differences in disease pathogenesis [18]. To explore the earliest defect(s) in the development of T2DM, the first-degree relatives (FDRs) of T2DM have been shown to represent an ideal mode, since they have high risk of developing T2DM [19]. Therefore, based on a cohort of FDRs, we employed the highly sensitive and specific methods for testing specific insulin and proinsulin and aimed to explore the relative roles of IR and *β*-cell dysfunction in the early stage of T2DM development in Chinese population and examine the usefulness of proinsulin and proinsulin-to-specific insulin ratio in detecting alterations of *β*-cell function.

## 2. Materials and Methods

### 2.1. Subjects

A total of 414 subjects aged between 30 and 70 years were recruited from outpatient clinic. All subjects were screened with 75 g oral glucose tolerance test (OGTT). Glucose tolerance status was defined according to the American Diabetes Association [20]; a subject was classified as having prediabetes (pre-DM) including the following: impaired fasting glucose (IFG): FBG ≥ 5.6 mmol/L to 7.0 mmol/L; impaired glucose tolerance (IGT): 2 h BG 7.8 mmol/L to 11.1 mmol/L. T2DM was diagnosed in patients with FBG ≥ 7.0 mmol/L or OGTT 2 h BG ≥ 11.1 mmol/L. FDRs of T2DM without diabetes were divided into two groups according to their OGTT status: 174 subjects with NGT (NGT-FDRs) and 55 with pre-DM (pre-DM-FDRs). In addition, 71 subjects with newly diagnosed T2DM (including 17 subject of FDRs) and 114 subjects of NGT without family history of T2DM (NGT-non-FDRs) were enrolled as T2DM control and normal control groups, respectively. In order to reduce the influence of life style factors, most of the subjects in normal control group were the spouses of their counterparts. Exclusion criteria included an acute illness three months before enrollment, previous diagnosis of diabetes, severe liver or kidney diseases, and any medications that would influence glucose metabolism. Written informed consent was obtained from all participants, and the study protocol was approved by local ethics committee.

### 2.2. Clinical Measurement and Laboratory Test

Weight, waist circumference (WC), and hip circumference were measured by trained field workers. Participants removed bulky clothing and shoes prior to measurements. WC was measured midway between the lowest rib and the top of the iliac crest. Hip circumference was measured around the widest portion of the buttocks. Weight was measured to the nearest 0.1 kg using a TANITA Body Composition Analyzer (Model: TBF-300A). BMI was calculated by body weight in kilogram divided by height square in meter. Measurements of right arm systolic blood pressure and diastolic blood pressure (SBP and DBP) were performed 3 times 10 minutes apart and the mean values of the latter two measurements were recorded.

Participants were instructed to refrain from excessive physical exercise and to take regular food three days before the test, with the amount of carbohydrate being no less than 200 grams. After fasting overnight (8–10 hours), a 2-hour OGTT test was performed on each subject. Blood glucose levels in the fasting state (FBG) and 2-hour BG were measured by hexokinase method. Subjects' renal and liver functions and blood lipids concentrations including triglycerides (TG), total cholesterol (TC), low-density lipoprotein cholesterol (LDL-C), and high-density lipoprotein cholesterol (HDL-C) were assayed using standard methods (Hitachi 7500 automatic biochemical analyzer, Japan). Plasma specific insulin and proinsulin levels were centrally measured in Peking Union Medical College Hospital using the in-house enzyme-linked immunosorbent assays (ELISA) [21]. The intra-assay and interassay coefficients of variation (CVs) were <4.1% and <7% for insulin and <5.8% and <10% for proinsulin, respectively. Insulin assay had no cross-reactivity to proinsulin (<0.05%) and vice versa.

### 2.3. Calculation and Definition

Insulin secretion was assessed by the HOMA-B model as fasting specific insulin (FSI; mIU/L) × 20/[FBG (mmol/L)-3.5] [4] and the fasting proinsulin (FPI; pmol/L) to specific insulin (pmol/L) ratio (FPI/SI). IR was evaluated by the HOMA-IR model calculated as HOMA-IR = FSI (mIU/L) × FBG (mmol/L)/22.5 and quantitative insulin sensitivity check index (QUICKI) = 1/(log⁡FSI + log⁡FBG) [5]. Fasting hyperinsulinemia, hyperproinsulinemia, high FPI/SI ratio, and IR were defined by ≥95th percentile of our healthy reference population; that is, FSI > 11.8 mIU/L, FPI > 9.8 pmol/L, FPI/SI > 25%, and HOMA-IR > 2.54. Metabolic syndrome (MetS) was diagnosed according to 2009 proposed harmonized criteria, if the subject had at least three of the following five components [22]: (1) central obesity: WC ≥ 90 cm for male and ≥ 80 cm for female; (2) IFG, IGT, or a diagnosis of diabetes; (3) elevated BP ≥ 130/85 mmHg; (4) HDL-C < 1.03 mmol/L in males and <1.29 mmol/L in females; and (5) TG ≥ 1.70 mmol/L.

### 2.4. Statistical Analysis

Data were presented as means ± SEM/SD for continuous parameters and percentage for categorical variables. Analysis of variance, general linear models (GLM), and *χ* ^2^ tests were used for descriptive statistics following Bonferroni or least significant difference (LSD)* Post hoc *comparison, respectively. Data were tested for normality of distribution and consequently HOMA-IR, specific insulin, and proinsulin were natural log (ln)-transformed to obtain normal distributions. General linear models were used to compare mean levels by adjusting for confounding factors. Linear correlation and stepwise regression analyses were used to assess the relationship between variables. Differences were considered significant if *p* < 0.05. All analysis was performed using SPSS version 19.0 (Chicago, IL, USA).

## 3. Results

### 3.1. The Basic Characteristics of the Study Groups

The four groups were similar in sex distribution. All parameters except waist circumference were comparable between the two NGT groups with or without family history of T2DM (Table 1). Meanwhile, the cardiometabolic parameters and the prevalence of hypertension, dyslipidemia, obesity, and MetS were increased with the severity of glucose intolerance, especially in newly diagnosed T2DM groups, among whom more than two-thirds of participants were identified as having MetS.

### 3.2. The Comparisons of IR and *β*-Cell Function among the Study Groups

The parameters reflecting IR and *β*-cell secretion function scattered largely across the study groups, except for QUICKI. Considering that age, BMI, and WC were not comparable among the four groups, GLM analysis was used to compare the means after adjusting for those variables. As shown in Table 2, fasting and 2 h SI levels reached their peaks in pre-DM stage and decreased in T2DM stage, but there was no significant deference in fasting SI between the two NGT groups from the FDRs or non-FDRs. In contrast, in comparison with the normal controls, NGT of FDRs showed significantly higher levels of proinsulin and PI/SI ratio both in fasting status and in 2-hour postglucose load. Moreover, fasting PI and PI/SI ratio were significantly increased from NGT-FGRs to pre-DM-FDRs and were highest in T2DM group (*p* < 0.001). Likewise, HOMA-IR showed the similar trend across the four groups (*p* < 0.001), while QUICKI was gradually decreased (*p* < 0.001) across the four groups. Compared with NGT-non-FDRs, geometric mean of HOMA-IR was 9% greater in NGT-FDRs, 33% greater in pre-DM-FDRs, and 74% greater in T2DM. HOMA-B, by contrast, was not different between NGT and pre-DM and decreased significantly only in T2DM group (by 70%, *p* < 0.001).

To further compare the IR and *β*-cell dysfunction among groups, we set the 95th percentile in normal group as the cutoff point to identify subjects with fasting hyperinsulinemia, hyperproinsulinemia, high FPI/TI ratio, and high HOMA-IR. As listed in Table 2, although there was no significant decrease of HOMA-B in subjects before diagnosis of T2DM, the prevalence of IR and the disproportionate hyperproinsulinemia were increasingly verified in the group of NGT-FDRs (2-fold to 4-fold) and both were about 10-fold higher in newly diagnosed T2DM group compared to control group, whereas the prevalence of hyperinsulinemia was peaked to 27.3% in pre-DM group and decreased to 17.1% in T2DM group. Meanwhile, 20% NGT-FDRs and 59% T2DM have high FPI/SI levels.

### 3.3. The Comparisons of *β*-Cell Function after Controlling for Insulin Sensitivity

It has been suggested that *β*-cell function should be assessed in the context of IR; we thus further adjusted the effect of insulin sensitivity with HOMA-IR or QUICKI by using GLM analysis. As shown in Table 3, the adjusted HOMA-B, albeit not being able to detect the difference between the two NGT groups, became more sensitive than its unadjusted model as it could show significant reduction (by 27% versus 6%; *p* < 0.001 versus *p* > 0.05) in pre-DM group, whereas a remarkable reduction was exhibited only in T2DM group (by 70%; *p* < 0.001) in unadjusted analyses (Table 2). On the other hand, when using fasting PI/SI ratio as a measure of *β*-cell dysfunction, progressive decline of *β*-cell function could be detected across the four groups and became more pronounced after adjusting for HOMA-IR (*p* < 0.001): relative to those in non-FDRs controls, the adjusted and unadjusted geometric means of FPI/SI were 1.5 versus 1.4-folds higher in NGT-FDRs, 2.0 versus 1.7-folds in pre-DM-FDRs, and 4.7 versus 3.5-folds in T2DM, respectively.

### 3.4. Bivariate Scatterplots for Distinguishing Glucose Abnormality or MetS

**Figure 1:**
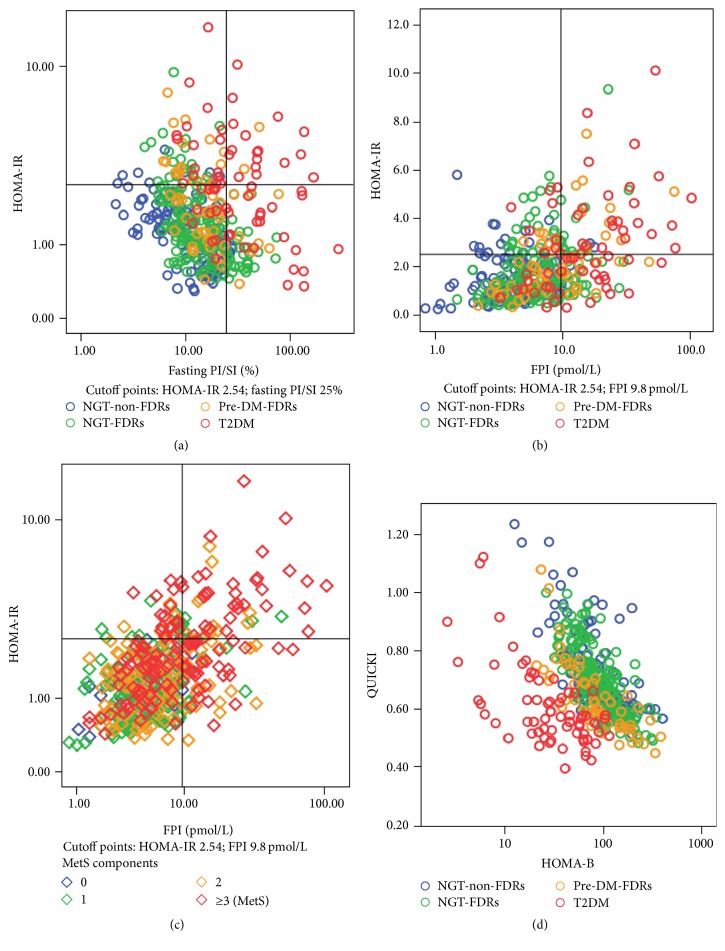
Scatterplots of measures for distinguishing abnormal glucose metabolism (panels (a), (b), and (d)) or metabolic syndrome (panel (c)). Notes: NGT-non-FDRs, normal glucose tolerance subjects with no family history of diabetes; NGT-FDRs, the first-degree relatives of T2DM with normal glucose tolerance; pre-DM-FDRs, first-degree relatives of T2DM with prediabetes; T2DM, type 2 diabetes mellitus; MetS, metabolic syndrome; HOMA-IR, homeostasis model assessment of insulin resistance; HOMA-B, homeostasis model assessment of beta-cell function; QUICKI, quantitative insulin sensitivity check index; PI, proinsulin; SI, specific insulin; PI/SI, proinsulin-to-specific insulin ratio.

The analysis above revealed that the trend of HOMA-IR, QUICKI, FPI, and FPI/SI ratio as well as HOMA-B became more significant in parallel with the worsening of glucose metabolism. To examine the potential bivariate associations of these indices in different glucose regulation groups, separated two-way scatterplots were performed for HOMA-IR versus FPI, HOMA-IR versus FPI/SI, and QUICKI versus HOMA-B. As shown in Figure 1(a), those with both high FPI/SI ratio and high HOMA-IR were exclusively identified as having abnormal glucose tolerance, which consisted of 10% pre-DM and 90% T2DM; while those with high FPI and high HOMA-IR (Figure 1(b)) consisted of 22% NGT-FDR, 20% pre-DM-FDR, and 54% T2DM. However, after further classifying with regard to MetS components, as shown in Figure 1(c), among those with both high FPI and HOMA-IR, 62% of subjects were MetS, and nearly 90% of subjects had at least two components of MetS. Those results indicated that FPI, combining with HOMA-IR, could specifically reflect the clustering characteristics of MetS components rather than the deterioration of glucose metabolism and thus appears to play a role in predicting cardiovascular risk. On the other hand, the scatterplot of QUICKI or 1/HOMA-IR (data not show) versus HOMA-B, as shown in Figure 1(d), could finely distinguish T2DM from other glucose groups and also showed the existence of a hyperbolic relationship between insulin sensitivity and secretion.

## 4. Discussion

As a high risk population of diabetes, FDRs have been studied extensively to clarify the mechanism of development of T2DM. However, the precise nature is still being debated. The major reasons might be attributed to the difficulties in accurately assessing insulin sensitivity and *β*-cell function and the different methods being used. Using specific insulin and proinsulin as the key biomarkers, the present study assessed IR and *β*-cell dysfunction from normal to impaired glucose tolerance offspring of Chinese T2DM patients. The major finding is that, even evaluated by the simple indices based solely on fasting status, both IR as determined by HOMA-IR and *β*-cell dysfunction by increased FPI/SI ratio appear to exist in the FDRs of T2DM when glucose tolerance was still in normal status. Furthermore, these changes deteriorated progressively in FDRs from NGT to pre-DM and became more serious in T2DM. Notably, FPI/SI, rather than HOMA-B, independent of IR, could detect *β*-cell dysfunction in early stage of glucose intolerance and thus is clearly superior to HOMA-B in this regard.

Our study has several implications. Firstly, to study the relative importance of IR and *β*-cell dysfunction and whether one defect precedes the other in the development of T2DM in Chinese population, we selected the FDRs of T2DM in different stage of glucose intolerance and their healthy counterparts without family history of diabetes for a comparative study. Meanwhile, to overcome the interference of circulating proinsulin, we used highly sensitive and specific method to detect “true” insulin levels instead of “immunoreactive insulin” in order to improve the accuracy of assessment [7–9]. Our results revealed that FDRs presented IR even if their glucose level were still in the normal range, as they showed higher HOMA-IR and lower QUICKI than their peers, which were in accordance with findings from some previous studies by using the gold methods of euglycemic clamp [23–25] but in contrast to other studies using similar surrogate indices made by nonspecific insulin measurements [26]. Furthermore, IR as assessed by HOMA-IR deteriorated progressively from NGT to pre-DM and T2DM. Those results suggest that IR not only is a key player in T2DM development but also contributes to the inheritance of T2DM in this Chinse population.

Secondly, our findings implied that *β*-cell defects may be more pronounced early in the pathogenesis of diabetes in Chinese population compared to previous reports [27]. In fact, determining *β*-cell failure is problematic in clinical practice; direct measures, such as the hyperglycemic clamp and the acute insulin response from an intravenous glucose tolerance test (IVGTT), are irrelevant for clinical use because of the laborious and expensive procedures [28, 29]. Thus, surrogate markers based on fasting blood samples are often used, namely, HOMA-B [7, 30], proinsulin levels, and the proinsulin-to-insulin ratio [10]. However, previous studies have demonstrated that subclinical *β*-cell defects could be identified even in insulin-sensitive nondiabetic offspring of patients by using hyperglycemia-hyperinsulinemic clamp, the OGTT, or the IVGTT [23, 24, 29], but decline of HOMA-B was not observed during early stage of abnormal glucose tolerance, leading to underestimate the magnitude of the -*β*-cell defect [31]. Similarly, in this study, when HOMA-B was used as a measure of *β*-cell function, we demonstrated that there was significant reduction (by ~70%) in newly diagnosed T2DM but not in pre-DM stage when analyses were not adjusted for insulin sensitivity, even in the fact that we calculated the HOMA index by using specific insulin to improve the methodological performance [30]. In contrast, when proinsulin-to-insulin ratio was used as a measure of *β*-cell dysfunction, even in the NGT stage, there was a significant increase in the FDRs of individuals with T2DM, irrespective of controlling for IR. Therefore, in this population, it appears that FPI/SI could reflect the early subclinical *β*-cell defect, suggesting that *β*-cell dysfunction plays a more important role in the development of T2DM than previously estimated from epidemiological studies using HOMA-B [27].

It has long been known that an increase in the amount of circulating proinsulin relative to circulating insulin implies the defective islet *β*-cell processing of the proinsulin molecule and is an early indicator of *β*-cell dysfunction [10, 32] and is subsequently used as a valuable measure to assess the effects of therapeutic interventions [13–15]; however, until recently, a number of the T2DM-associated genetic variants known to impact *β*-cell function have been shown to be associated with proinsulin to insulin ratio [16, 17], highlighting the importance of proinsulin as a direct measure of *β*-cell dysfunction. Therefore, we chose proinsulin based indices of *β*-cell function as they include possible genetic effects. Up to now, there are limited studies that compare proinsulin as well as proinsulin-to-insulin ratio with other measures of *β*-cell function in offspring of T2DM [10], mostly conducted in the high risk populations such as Mexican-Americans, Hispanic-Americans, and European populations [33–35], but not all [34] have reported similar findings. To our knowledge, there is no report on Chinese population. In this study, we found that elevated fasting PI/SI levels were already present among the FDRs with NGT in comparison with controls; further, FPI/SI increased progressively with decreased glucose tolerance, and these differences became more significant when controlling for IR. However, with respect to proinsulin or PI/SI levels at 2 hours of an OGTT, we found the difference among the four groups was not so significant as their fasting levels, which was slightly consistent with the finding of a large population-based study in Finnish men [35]. Nevertheless, our study provides evidence that, in this Chinese population, fasting PI/SI could not only detect the early defects of *β*-cell function but also reflect the degree of *β*-cell dysfunction in the context of IR in different stages of glucose tolerance, thus providing a sensitive and simple mean for evaluating *β*-cell dysfunction [10].

Thirdly, it has been reported that, in normal subjects, only 10% to 20% of secreted insulin comprises proinsulin and/or its conversion intermediates, but the proportion can reach 50% in T2DM [10]. However, due to the fact that conventional insulin assay has cross-reactivity with proinsulin, there is a debate as to whether hyperinsulinemia or hyperproinsulinemia exists in newly diagnosed T2DM [10]. Moreover, it has been speculated that elevated proinsulin levels are also indicative of IR and cardiovascular risk [10, 36]. To our knowledge, rare studies have analyzed proinsulin and the proinsulin-to-insulin ratio in Chinese population. In this study, based on specific measurements of insulin and proinsulin, we not only set up their normal reference levels but also compared their relationship with the classical HOMA index/QUICKI by scatterplot analysis to find their different significance in evaluating abnormal glucose metabolism and cardiovascular risk. Our study found that, in the FDRs of T2DM, hyperproinsulinemia (defined by fasting proinsulin ≥9.8 pmol/L) has been identified as 20% in NGT, 45% in pre-DM group, and more than 2/3 in T2DM group, while true hyperinsulinemia was 27% in pre-DM group and only 17% in T2DM group. Thus, at least in this Chinese population, most of the newly diagnosed T2DM were presented with hyperproinsulinemia rather than true hyperinsulinemia. Furthermore, based on multivariate analysis, we also demonstrated that fasting PI could independently predict IR (data not shown). Moreover, glucose abnormality could be specifically classified based on the scatterplot of HOMA-IR versus FPI/SI, while the clustering of MetS components could be well identified based on HOMA-IR versus FPI. Our findings suggest that combination use of those measures might have different values for disease prediction as well as selections of appropriate therapy and monitoring of treatment success. In line with us, Pfützner and Forst [10] proposed to choose treatment regimen and conduct treatment evaluation based on specific insulin and proinsulin levels in clinical practice.

However, our study also has some limitations. Firstly, although surrogate markers such as HOMA-IR, fasting proinsulin, and PI/SI ratio appeared to be valuable in current study, due to lack of universal standard methods to measure insulin and proinsulin, there are no universally agreed at “normal ranges” yet [10, 37]. Consequently, our finding could not be simply generalized to the other population. For example, what is the appropriate cut-point of HOMA-IR for identifying IR? It was reported that HOMA-IR ≥ 2 could be judged as IR when testing by the specific insulin method [10, 38], while a widely adopted cutoff was 2.60 [37], which was very consistent with our finding of 2.54 based on ≥95th percentile of our normal group. Since the reliability of HOMA index is absolutely dependent on the accuracy of insulin assay, comparable measures of insulin sensitivity and secretion for practical clinical care might not be realized until those assays are well standardized [36]. Secondly, we did not compare those simple measures directly with the gold standards of clamp test. Finally, although we have carefully chosen the controls, we could not determine the causal relationship due to the cross-sectional nature of the study; therefore, our findings will require further verification in a longitudinal analysis.

In conclusion, based on a well-characterized cohort of FDRs, we observed that both IR (as evaluated by HOMA-IR using specific insulin) and *β*-cell dysfunctions (disproportionately elevated proinsulin) existed in the offspring of Chinese patients with T2DM when glucose tolerance was still in normal status. HOMA-IR could discriminate the progressive changes of IR during the deterioration of glucose tolerance, while fasting PI/SI, but not HOMA-B, independent of IR, could differentiate the decline of *β*-cell function among different glucose tolerance stages, thus providing an additional sensitive measure of *β*-cell dysfunction.

## Figures and Tables

**Table 1:** The basic clinical and biochemical characteristics of the study groups.

	Non-FDRs NGT	FDRs		T2DM	*p* value
		NGT	Pre-DM		
Male/female (*n*)	54/60	70/104	23/32	38/33	0.245
Age (years)	44.2 ± 8.2	43.5 ± 8.2	47.8 ± 7.0^*∗*#^	49.2 ± 9.2^*∗∗*##^	<0.001
BMI (kg/m^2^)	24.1 ± 3.0	24.5 ± 3.2	25.7 ± 3.1^*∗∗*#^	26.4 ± 2.5^*∗∗*##^	<0.001
WC (cm)	80.7 ± 10.6	83.7 ± 9.6^*∗*^	87.6 ± 9.4^*∗∗*#^	91.8 ± 5.9^*∗∗*#^	<0.001
Waist-to-hip ratio	0.83 ± 0.07	0.84 ± 0.07	0.86 ± 0.06^*∗∗*#^	0.87 ± 0.06^*∗∗*#^	0.002
SBP (mmHg)	116.6 ± 13.4	114.1 ± 13.7	120.8 ± 18.1^##^	126.7 ± 11.9^*∗∗*##†^	<0.001
DBP (mmHg)	77.0 ± 8.8	74.9 ± 8.6	76.7 ± 8.7	77.7 ± 7.3^##^	0.006
FBG (mmol/L)	4.9 ± 0.5	4.9 ± 0.4	5.6 ± 0.6^*∗∗*##^	8.6 ± 2.6^*∗∗*##††^	<0.001
2 h BG (mmol/L)	5.4 ± 1.2	5.7 ± 1.1	8.7 ± 1.0^*∗∗*##^	15.0 ± 4.1^*∗∗*##††^	<0.001
TC (mmol/L)	4.8 ± 0.9	4.8 ± 0.8	4.7 ± 1.3	4.1 ± 2.0^*∗∗*,##††^	0.010
TG (mmol/L)	1.6 ± 1.3	1.6 ± 1.6	2.5 ± 2.3^*∗∗*##^	3.7 ± 2.0^*∗∗*##††^	<0.001
HDL-C (mmol/L)	1.3 ± 0.4	1.4 ± 0.3	1.2 ± 0.4^*∗*#^	1.1 ± 0.2^*∗∗*##^	<0.001
LDL-C (mmol/L)	2.8 ± 0.7	2.9 ± 0.7	3.0 ± 0.7	3.1 ± 1.0^*∗*^	0.093
ALT (IU/L)	21.2 ± 13.9	20.8 ± 16.6	21.5 ± 12.7	32.3 ± 23.9^*∗*##†^	0.015
AST (IU/L)	22.0 ± 8.3	21.8 ± 8.7	23.6 ± 9.0	28.7 ± 12.5^*∗∗*##^	0.004
Uric acid (*μ*mol/L)	297 ± 96	305 ± 94	307 ± 87	330 ± 97	0.521
Creatine (*μ*mol/L)	62.6 ± 14.4	61.2 ± 12.1	60.3 ± 14.3	62.8 ± 17.1	0.725
Hypertension (%)	18.5	19.6	30.6^*∗*#^	56.5^*∗∗*#†^	<0.001
Dyslipidemia (%)	28.3	29.5	44.9^*∗*#^	78.7^*∗∗*##††^	<0.001
Overweight or obesity (%)	38.6	38.9	58.2^*∗*#^	66.2^*∗∗*#†^	<0.001
MetS (%)	5.1	7.8	50.9^*∗∗*##^	71.7^*∗∗*##†^	<0.001

Notes: data are means ± SD or frequency distribution. *p-*values were obtained from ANOVA or *χ* ^2^ tests for overall comparison across the four groups.

^*∗*^ *p* < 0.05, ^*∗∗*^ *p* < 0.01, compared with NGT-non-FDRs

^#^ *p* < 0.05,  ^##^ *p* < 0.01, compared with NGT-FDRs

^†^ *p* < 0.05, ^††^ *p* < 0.01, compared with pre-DM-FDRs

Abbreviations: NGT-non-FDRs, normal glucose tolerance subjects with no family history of diabetes; NGT-FDRs, the first-degree relatives of T2DM with normal glucose tolerance; pre-DM-FDRs, first-degree relatives of T2DM with pre-diabetes; T2DM, type 2 diabetes mellitus; BMI: body mass index; WC: waist circumference FBG: fasting blood glucose; SBP: systolic blood pressure; DBP: diastolic blood pressure; TG: triglycerides; TC: total cholesterol; LDL-C: low-density lipoprotein cholesterol; HDL-C: high-density lipoprotein cholesterol; AST: aspartate transaminase; ALT: alanine transaminase: MetS, metabolic syndrome.

**Table 2:** Comparisons of insulin resistance and *β*-cell function in different groups.

	Non-FDRs NGT	FDRs		T2DM	*p* value
		NGT	Pre-DM		
^a^Ln HOMA-IR	0.21 ± 0.05	0.30 ± 0.04^*∗*^	0.5 ± 0.07^*∗∗*##^	0.77 ± 0.0 7^*∗∗*##†^	<0.001
QUICKI	0.72 ± 0.01	0.69 ± 0.01^*∗*^	0.65 ± 0.02^*∗∗*#^	0.62 ± 0.01^*∗∗*##^	<0.001
Ln HOMA-B	4.45 ± 0.06	4.51 ± 0.05	4.39 ± 0.09	3.24 ± 0.08^*∗∗*##††^	<0.001
Ln fasting SI (mIU/L)	1.74 ± 0.05	1.82 ± 0.04	1.99 ± 0.07^*∗∗*#^	1.75 ± 0.06^†^	0.02
Ln 2 h SI (mIU/L)	3.36 ± 0.08	3.57 ± 0.06^*∗*^	4.24 ± 0.11^*∗∗*##^	3.53 ± 0.10^††^	<0.001
Ln fasting PI (pmol/L)	1.39 ± 0.06	1.86 ± 0.04^*∗∗*^	2.29 ± 0.08^*∗∗*#^	2.69 ± 0.08^*∗∗*##††^	<0.001
Ln 2 h PI (pmol/L)	3.28 ± 0.07	3.76 ± 0.06^*∗∗*^	4.36 ± 0.10^*∗∗*##^	3.87 ± 0.09^*∗∗*††^	<0.001
Ln fasting PI/SI	2.29 ± 0.06	2.67 ± 0.05^*∗∗*^	2.84 ± 0.09^*∗∗*#^	3.56 ± 0.08^*∗∗*##††^	<0.001
Ln 2 h PI/SI	2.56 ± 0.07	2.80 ± 0.06^*∗*^	2.75 ± 0.10	2.92 ± 0.10^*∗∗*##††^	<0.01
Hyperproinsulinemia (%)	4.9	21.3^*∗∗*^	45.5^*∗∗*##^	64.5^*∗∗*##††^	<0.001
High fasting PI/SI (%)	4.5	19.5^*∗∗*^	24.5^*∗∗*#^	58.6^*∗∗*##††^	<0.001
Hyperinsulinemia (%)	4.5	11.5^*∗*^	27.3^*∗∗*##^	17.1^*∗*#†^	<0.001
Insulin resistance (%)	5.4	17.6^*∗∗*^	33.8^*∗∗*##^	54.6^*∗∗*##††^	<0.001

Notes: data are means ± SEM or percentages (%) with adjustment of age, sex, and waist circumference.

^*∗*^ *p* < 0.05 and ^*∗∗*^ *p* < 0.01, compared with NGT-non-FDRs.

^#^ *p* < 0.05 and  ^##^ *p* < 0.01, compared with NGT-FDRs.

^†^ *p* < 0.05 and ^††^ *p* < 0.01, compared with pre-DM-FDRs.

^a^Ln: natural log-transformed.

NGT-non-FDRs, normal glucose tolerance subjects with no family history of diabetes; NGT-FDRs, the first-degree relatives of T2DM with normal glucose tolerance; pre-DM-FDR, first-degree relatives of T2DM with prediabetes; T2DM, type 2 diabetes mellitus; HOMA-IR, homeostasis model assessment of insulin resistance; HOMA-B, homeostasis model assessment of beta-cell function; QUICKI, quantitative insulin sensitivity check index; PI, proinsulin; SI, specific insulin; PI/SI, proinsulin-to-specific insulin ratio.

**Table 3:** Comparisons of *β*-cell function after adjusting for HOMA-IR.

	Non-FDRs NGT	FDRs		T2DM	*p* value
		NGT	Pre-DM		
^a^Ln HOMA-B	4.56 ± 0.05	4.59 ± 0.04	4.25 ± 0.07^*∗∗*##^	2.94 ± 0.06^*∗∗*##††^	<0.001
^a^Ln fasting PI/SI	2.21 ± 0.06	2.62 ± 0.04^*∗∗*^	2.91 ± 0.08^*∗∗*#^	3.76 ± 0.07^*∗∗*##††^	<0.001

Notes: data are means ± SEM with adjustment for age, sex, waist circumference, and HOMA-IR.

^a^Ln: natural log-transformed.

^*∗*^ *p* < 0.05 and ^*∗∗*^ *p* < 0.01, compared with NGT-non-FDRs using Bonferroni correction.

^#^ *p* < 0.05 and  ^##^ *p* < 0.01, compared with NGT-FDRs.

^†^ *p* < 0.05 and ^††^ *p* < 0.01, compared with pre-DM-FDRs.

NGT-non-FDRs, normal glucose tolerance subjects with no family history of diabetes; NGT-FDRs, the first-degree relatives of T2DM with normal glucose tolerance; pre-DM-FDRs, first-degree relatives of T2DM with prediabetes; T2DM, type 2 diabetes mellitus; HOMA-IR, homeostasis model assessment of insulin resistance; HOMA-B, homeostasis model assessment of beta-cell function; PI/SI, proinsulin-to-specific insulin ratio.
